# Cognitive training effects in mild cognitive impairment with and without amyloid pathology

**DOI:** 10.1590/1980-5764-DN-2025-0398

**Published:** 2026-08-24

**Authors:** Nadia Aparecida Bossa, Geraldo Busatto, Ricardo Nitrini, Sonia Maria Dozzi Brucki, Eliane Correa Miotto

**Affiliations:** 1 Universidade de São Paulo, Faculdade de Medicina, Departamento de Neurologia, São Paulo SP, Brazil. Universidade de São Paulo Faculdade de Medicina Departamento de Neurologia SP São Paulo Brazil; 2 Universidade de São Paulo, Faculdade de Medicina, Departamento de Psiquiatria, São Paulo SP, Brazil. Universidade de São Paulo Faculdade de Medicina Departamento de Psiquiatria SP São Paulo Brazil

**Keywords:** Mild Cognitive Impairment, Cognitive Training, Memory,Episodic, Alzheimer Disease, Neuronal Plasticity, Neuropsychological Tests, Comprometimento Cognitivo Leve, Treino Cognitivo, Memória Episódica, Doença de Alzheimer, Plasticidade Neuronal, Testes Neuropsicológicos

## Abstract

Imagery-based cognitive training (CT) is a promising strategy for individuals with amnestic mild cognitive impairment (aMCI). However, there is a lack of studies investigating its differential impact in aMCI with and without amyloid pathology.

**Objective:**

To investigate the effects of CT based on mental imagery on episodic memory in individuals with amyloid-positive aMCI (Aβ+), amyloid-negative aMCI (Aβ-), and healthy controls (HC).

**Methods:**

Fifty-five participants were included: Aβ+aMCI (n=19), Aβ-aMCI (n=20), and HC (n=16), aged 51–89 years, with more than 6 years of education. Amyloid status was confirmed by 11C-PiB PET. All participants completed pre- and post-training neuropsychological assessments and attended six individual online CT sessions. Performance was assessed using the logical memory test (LM), Rey Auditory Verbal Learning Test (RAVLT), and generalization tasks (GT I–II).

**Results:**

Data were analyzed using the Shapiro-Wilk, Levene, Wilcoxon, ANOVA, Kruskal-Wallis, and Bonferroni tests. Significant improvements in immediate episodic memory (LM I, RAVLT A1–A5) were observed in the Aβ+ and Aβ-groups, particularly in the Aβ-aMCI group. Delayed recall (LM II, RAVLT A7) increased, although without statistical significance. All groups showed gains in generalization tasks, supporting the benefit of CT.

**Conclusion:**

Unimodal CT based on mental imagery promoted gains in immediate episodic memory across all groups. Although delayed recall gains were not statistically significant, the strategy was internalized and generalized. Similar responsiveness in Aβ+ and Aβ-groups suggest preserved compensatory mechanisms despite amyloid pathology. These findings suggest more robust effects at the level of encoding than consolidation, which may vary according to task characteristics and underlying neural integrity.

## RESUMO

O treino cognitivo (TC) baseado em imagens mentais é uma estratégia promissora para indivíduos com comprometimento cognitivo leve amnéstico (CCLa). Entretanto, há uma escassez de estudos que investiguem seu impacto diferencial em indivíduos com CCLa com e sem patologia amiloide.

**Objetivo:**

Investigar os efeitos do TC baseado em imagens mentais sobre a memória episódica em indivíduos com CCLa amiloide-positivo (Aβ+), amiloide-negativo (Aβ-), e controles saudáveis (CS).

**Métodos:**

Foram incluídos 55 participantes: CCLaAβ+ (n=19), CCLaAβ-n=20) e CS (n=16), com idades entre 51 e 89 anos e mais de 6 anos de escolaridade. O *status* amiloide foi confirmado por tomografia por emissão de pósitrons (PET) com 11C-PiB. Todos os participantes foram submetidos a avaliações neuropsicológicas prée pós-treino e participaram de seis sessões individuais de TC *online.* O desempenho foi avaliado utilizando o Teste de Memória Lógica (ML), o teste de Aprendizagem Auditivo-Verbal de Rey (RAVLT) e as Tarefas de Generalização (TG I–II).

**Resultados:**

Os dados foram analisados utilizando os testes de Shapiro– Wilk, Levene, Wilcoxon, ANOVA, Kruskal–Wallis e Bonferroni. Foram observadas melhoras significativas na memória episódica imediata (ML I, RAVLT A1–A5) nos grupos Aβ+ e Aβ-, particularmente no grupo CCLaAβ-. A evocação tardia (ML II, RAVLT A7) aumentou, embora sem significância estatística. Todos os grupos apresentaram ganhos nas tarefas de generalização, corroborando o benefício do TC.

**Conclusão:**

O TC unimodal baseado em imagens mentais promoveu ganhos na memória episódica imediata em todos os grupos. Embora os ganhos na evocação tardia não tenham sido estatisticamente significativos, a estratégia foi internalizada e generalizada. A responsividade semelhante nos grupos Aβ+ e Aβ-sugere a preservação de mecanismos compensatórios apesar da patologia amiloide. Esses achados sugerem efeitos mais robustos no nível da codificação do que na consolidação, os quais podem variar de acordo com as características da tarefa e a integridade neural subjacente.

## INTRODUCTION

Mild Cognitive Impairment (MCI) is a clinical condition characterized by measurable cognitive decline that exceeds expectations for age and educational level but does not significantly interfere with activities of daily living^1^. Its amnestic subtype (aMCI), which primarily affects episodic memory, is considered the phenotype with the highest risk of progression to Alzheimer disease (AD), particularly when associated with pathological biomarkers.

In recent years, Cognitive Training (CT) has emerged as a promising non-pharmacological strategy to promote neuroplasticity and preserve cognitive functions in individuals with MCI. Several clinical trials have shown that cognitive interventions can lead to improvements in memory, attention, language, and functional performance in this population^2,3^. However, most of these studies have not stratified participants based on biological biomarkers, such as cerebral β-amyloid deposition, thus limiting the translational value of the findings. This methodological gap prevents the identification of whether the observed cognitive benefits are comparable between individuals with MCI due to Alzheimer’s pathology (Aβ+) and those with other etiologies (Aβ-).

Since the publication of the biological framework proposed by Jack et al. in 2018^4^, and its update in 2024^5^, the diagnostic definition of AD has incorporated biomarker-based criteria, even in the absence of overt dementia symptoms. According to this model, the presence of cerebral amyloid (A), pathological tau (T), and neurodegeneration (N) constitutes the diagnostic foundation of AD, enabling a more accurate preclinical and etiological classification.

Recent systematic reviews^6,7^ emphasize that most CT studies in MCI fail to incorporate amyloid biomarker screening into their inclusion criteria. Moreover, longitudinal evidence confirms that Aβ+ individuals with amnestic MCI are significantly more likely to progress to dementia than their Aβ-counterparts^8^, reinforcing the importance of biomarker-based stratification in intervention research.

In this context, the present study aimed to address this critical gap by comparing the effects of a CT protocol based on mental imagery strategies involving brief narrative news texts with ecological validity, presented with and without visual support, across three groups:

- Aβ+ amnestic MCI (with amyloid pathology) (Aβ+aMCI);
- Aβ-amnestic MCI (without amyloid pathology) (Aβ-aMCI); and
- Cognitively healthy controls (HC).

This approach is expected to provide empirical evidence to support the refinement of CT protocols and the development of more personalized intervention strategies for both clinical and preclinical populations.

It is worth noting that although some studies have employed structured cognitive strategies, such as visual imagery, in individuals with aMCI, particularly those conducted by Belleville et al.^9^ and Hampstead et al.^10^, relevant methodological differences remain. For instance, Belleville et al.^9^ applied a multimodal protocol combining imagery, semantic categorization, and name-face associations in clinically diagnosed aMCI participants without biomarker stratification, limiting conclusions regarding the underlying etiology. Similarly, Hampstead et al.^10^ investigated the effects of mental imagery training on episodic spatial memory but did not incorporate ecological stimuli or biomarker-based grouping. Thus, few studies to date have employed unimodal CT protocols within ecologically valid frameworks while simultaneously accounting for participants’ biological profiles. The present study represents a conceptual and methodological advancement by integrating these three elements: specificity of the cognitive strategy, ecological relevance of the stimulus material, and stratification based on amyloid biomarker status^11^.

## METHODS

### Study design

This study constituted a secondary analysis of data derived from a prospective, double-blind clinical trial involving individuals with multiple-domain aMCI, with and without amyloid pathology, assessed using positron emission tomography (PET) imaging with the 11C-PIB tracer. The original trial was registered at ClinicalTrials. gov (NCT03263247) and approved by the Research Ethics Committee of the School of Medicine of Universidade de São Paulo (CAPPesq No. 11264).

The original study employed a double-blind design at multiple stages of data collection and intervention. Nuclear medicine physicians responsible for amyloid PET interpretation were blinded to participants’ clinical diagnoses. Likewise, neuropsychologists conducting the neuropsychological assessments were blinded to participants’ amyloid status and clinical classification. Neuropsychologists responsible for administering the cognitive training protocol were different from those conducting the assessments and were also blinded to participants’ biomarker status, neuropsychological results, and diagnostic classification throughout the intervention. These procedures were adopted to minimize expectancy effects and observer bias during both assessment and training phases.

The present study retrospectively evaluated the efficacy of a CT program based on mental imagery in a subsample of individuals with aMCI and HC. The intervention was grounded in established neuropsychological rehabilitation frameworks, which conceptualize cognitive training as a structured set of interventions aimed at improving or maintaining cognitive functions through targeted strategies. The training protocol emphasized the use of mental imagery as a mnemonic strategy to enhance encoding processes, particularly in tasks involving associative and episodic memory. This approach is consistent with prior evidence demonstrating that strategy-based interventions can modulate memory performance and support cognitive functioning in clinical populations^12^.

PET/CT scans were acquired using a Discovery-710 PET/CT scanner (GE Healthcare, Milwaukee, USA). A skull CT scan was performed prior to PET acquisition to enable attenuation correction and anatomical localization, using 120 kVp and 70 mA (or 140 mA, with 0.5 s per rotation). Further details regarding the radiochemical synthesis of [11C]PIB can be found in previously published studies^13,14^.

The classification of participants as Aβ+ or Aβ-followed the criteria of Coutinho et al.^14^. Two board-certified nuclear medicine physicians with more than five years of experience independently evaluated all [11C] PIB-PET scans, blinded to participants’ diagnoses. Scans were classified as positive when increased [11C] PIB uptake was observed in the cortical gray matter, resulting in reduced gray-to-white matter contrast, in at least two of the following six regions: frontal, temporal, lateral parietal, anterior cingulate, posterior cingulate cortices, and precuneus. Scans showing strong diffuse uptake within a single large cortical region were also classified as positive. Scans were classified as negative if uptake was restricted to white matter without substantial cortical gray matter involvement.

Following the visual assessment, both physicians performed a semiquantitative analysis using the 3D-SSP method (Cortex ID Suite software, GE Healthcare), developed specifically for the clinical interpretation of amyloid PET imaging. Standardized uptake value ratios (SUVr) were calculated for cortical regions normalized to cerebellar gray matter. A composite SUVr cutoff of 1.42 was applied for Aβ+ classification, complementing the visual evaluation. In cases of persistent discordance between the two readers after the 3D-SSP analysis, a consensus interpretation was obtained.

### Participants

A total of 55 participants (aged 51–89 years) were included. Individuals with aMCI were classified according to Petersen et al.^15^, and the sample also included HC. Participants were distributed into three groups:

- Aβ+aMCI: amnestic MCI with β-amyloid (n=19);
- Aβ-aMCI: amnestic MCI without β-amyloid (n=20);
- HC: healthy controls (n=16).

Participants were not randomized to diagnostic groups. Group allocation was determined by clinical diagnosis and amyloid PET status, resulting in three naturally occurring groups.

Participants with aMCI were recruited from multiple sources:

- The database of the research project approved by CAPPesq 0064/11;
- Patients followed at the outpatient clinic of the Cognitive Neurology and Behavior Group (GNCC), Hospital das Clínicas, School of Medicine of Universidade de São Paulo (HC/FMUSP); and
- The Reference Center for Cognitive Disorders (Centro de Referência em Distúrbios Cognitivos – CEREDIC) and the São Paulo Research Foundation (Fundação de Amparo à Pesquisa do Estado de São Paulo – FAPESP) projects 2012/50329-6 and 2014/50873-3.

When necessary, additional participants were recruited through public advertisement.

All participants were retired and were evaluated by neurologists specialized in cognitive neurology. Cognitive assessments were conducted by trained neuropsychologists. Individuals with aMCI had previously undergone amyloid biomarker assessment using Pittsburgh Compound B PET (11C-PiB PET), which allowed classification into Aβ+ and Aβ-groups. Healthy controls aged 60 years old or older were recruited from the community.

A total of 83 volunteers were screened for participation in the study. Of these, 13 did not meet the inclusion criteria, 11 declined participation, and 4 did not complete the cognitive training protocol and were therefore excluded from the analysis. The present study represents a secondary analysis of an ongoing clinical trial and includes only participants who completed all study procedures and for whom complete data were available at the time of this first analysis. Accordingly, the final sample consisted of 55 participants.

Given the longitudinal nature of the study and the complexity of the assessment protocol, participant attrition was expected. The study required neurological evaluation, amyloid PET imaging, comprehensive neuropsychological assessment, completion of the cognitive training protocol, and post-intervention reassessment, with the interval between recruitment and completion of all study procedures often extending over several months and, in some cases, exceeding one year. As commonly observed in longitudinal studies involving older adults with cognitive impairment, participant attrition may result from intercurrent medical conditions, hospitalization, clinical deterioration, relocation, transportation difficulties, caregiver-related constraints, or inability to complete all stages of the protocol. The study remains ongoing, and longitudinal follow-up continues beyond the sample included in the present analysis.

### Inclusion criteria

Presence of subjective memory complaint; performance ≥1.5 standard deviation (SD) below normative values on episodic memory and other cognitive domains^15^, as assessed by standardized neuropsychological tests, including the Rey Auditory Verbal Learning Test (RAVLT)^16^and the Logical Memory (LM) I and II subtests of the Wechsler Memory Scale-Revised (WMS-R)^17^; functional independence as measured by the Pfeffer Functional Activities Questionnaire (FAQ)^18^; absence of dementia; and amyloid status confirmed by [11C]PIB-PET.

Healthy controls also underwent the same neuropsychological and functional assessments to confirm the absence of cognitive impairment. The same neuropsychological instruments were used both for group classification at baseline and as outcome measures in pre- and post-training assessments.

### Exclusion criteria

Major psychiatric disorders; neurodegenerative diseases (except MCI); use of psychotropic drugs; sensory deficits affecting task performance; <6 years of education.

### Procedures

Before and after the cognitive training protocol, all participants from the three groups (Aβ+, Aβ-, and healthy controls) underwent the same standardized neuropsychological and clinical assessment procedures using the following instruments:

- Wechsler Adult Intelligence Scale-III (WAIS-III)^19^: Used to assess overall intellectual functioning (IQ).
- RAVLT^16^:
  - A1–A5: Assesses immediate recall.
  - A7: Assesses delayed recall.
- LM I and II (WMS-R)^17^: The first assesses immediate verbal recall, and the second assesses delayed recall.
- Trail Making Test, Parts A and B^20^: Assesses visual attention, processing speed, and cognitive flexibility.
- Rey-Osterrieth Complex Figure Test^21^: Evaluates visual memory and perceptual organization.
- Semantic and Phonemic Verbal Fluency Tests^22^: Measure lexical access, retrieval speed, and verbal executive control.
- Stroop Test – Victoria Version^23^: Assesses selective attention and inhibitory control over irrelevant responses (duration: approximately 10 minutes).
- Clock Drawing Test^24^: Assesses visuospatial organization, planning, and constructive praxis through the spontaneous drawing of a clock face.
- FAQ^18^: Evaluates functional independence in instrumental activities of daily living (IADLs), providing a measure of autonomy and daily functional status.

### Cognitive training protocol structure

The CT was structured into six individual online sessions, followed by a follow-up assessment. The sessions were conducted by a neuropsychologist, with a frequency of one session per week, each lasting approximately 90 minutes, and delivered synchronously via a virtual platform (Google Meet or Zoom). All sessions were conducted individually, with no group-based intervention components.

The protocol was carefully designed based on principles of mental imagery encoding and included progressively structured tasks integrating external visual imagery (through drawing-based encoding) and internally guided mental imagery, with the goal of facilitating the acquisition, consolidation, and generalization of the strategy^12^.

The training protocol followed a structured and progressive approach, with tasks increasing in complexity across sessions. During the initial sessions, participants were instructed to transform verbal stimuli (words and short sentences) into external visual representations through drawing, with step-by-step guidance provided by the neuropsychologist. In intermediate stages, participants were guided to construct mental images based on increasingly complex verbal material (*e.g*., sentences and short narratives), integrating visual and semantic elements.

In later sessions, the protocol emphasized internally generated mental imagery without external visual support, requiring participants to encode and retrieve information using self-generated visualization strategies. Tasks were standardized across participants, and performance was monitored during each session, with progression based on accuracy and recall performance.

**Figure 1:**
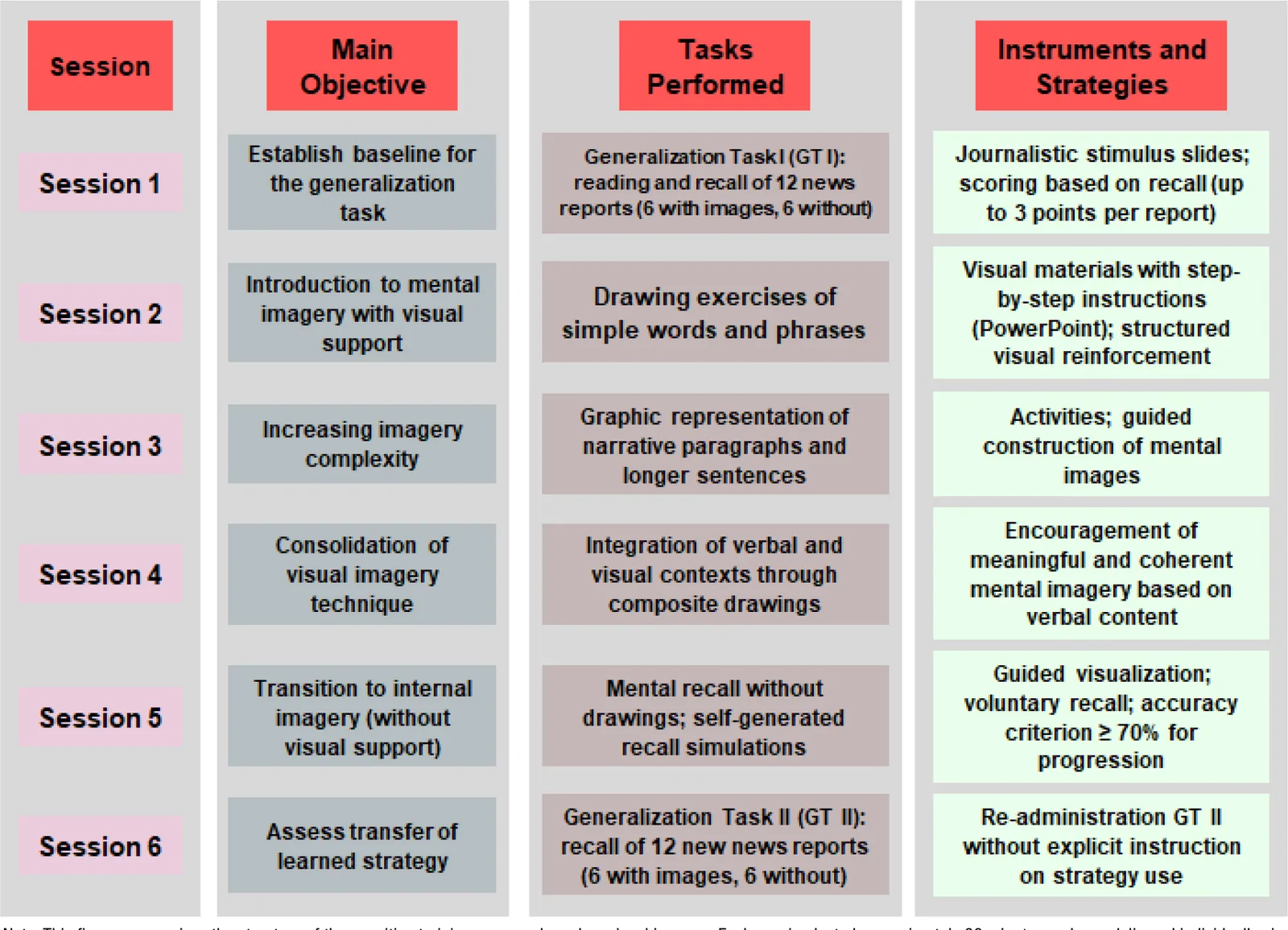
Structure of the Visual Imagery-Based Cognitive Training Program

Note: This figure summarizes the structure of the cognitive training program based on visual imagery. Each session lasted approximately 90 minutes and was delivered individually via synchronous online meetings. The training comprised progressively complex tasks, beginning with extedrnal visual imegery (drawing words and phrases) and transitioning to internal imagery (mental visualization), including generalization stages without explicit intructions. Generalization tasks were applied at three timepoints: at baseline (GT I), post-training (GT II), using journalistic materials either accompanied by images or presented as text only.

Source: The authors.

The stimuli consisted of controlled verbal materials (words, sentences, and short texts) and ecologically valid content (journalistic reports), presented either in verbal-only or dual-modality (image+text) formats. All training procedures were conducted during supervised sessions, with no structured home-based training or between-session practice prescribed (Figure 1).

### Instruments

#### Standardized neuropsychological assessment

The instruments from the original study used to assess the cognitive functions targeted in the present research are described below.

Participants underwent a comprehensive neuropsychological assessment before and after the cognitive training protocol. These assessments, including the RAVLT and LM tests, were administered at baseline (pre-training) and after completion of the training protocol, independently of generalization tasks (GT) I and II, which were conducted within specific training sessions. The same neuropsychological battery was used for group classification at baseline and for pre- and post-training assessments. For the purposes of the present study, analyses focused on episodic memory measures (RAVLT and LM) and GT performance.

Thus, the following instruments from the original neuropsychological battery were used:

- RAVLT^16^:
  - A1–A5: immediate recall of episodic memory;
  - A7: delayed recall of episodic memory.
- LM I and II-WMS^17^: assesses immediate (LM I) and delayed (LM II) recall of narrative episodic memory.

#### Generalization tasks

GTs measured near-transfer effects of cognitive training using a news recall paradigm simulating real-life memory demands^8^. Each version followed the same structure:

- 12 news reports not previously read by participants, equally divided into:
  - 6 with image and text (dual modality);
  - 6 with text only (verbal modality).

Participants were instructed to read each report and freely recall its content, without explicit strategic guidance.

Scoring was performed in real time by the neuropsychologist (standardized correction rubric), assigning 0 to 3 points per report based on the amount and accuracy of recalled information.

Each task included:

- Immediate recall (maximum: 36 points);
- Delayed recall (maximum: 36 points);
- Total per task: up to 72 points.

GT were applied at two time points:

- GT I – Session 1 (baseline);
- GT II – Session 6 (post-training).

The task assessed participants’ ability to spontaneously apply the trained strategy to novel content, indicating generalization and retention effects beyond the stimuli used during training. The analysis focused on performance in GT I and GT II, with GT I as the baseline measure and GT II capturing immediate generalization effects post-training.

#### Statistical analysis

The statistical analyses aimed to evaluate both with-in-group (pre- *vs.* post-CT) and between-group (Aβ+, Aβ-, and HC) effects resulting from the visual imagery-based CT protocol. Additionally, clinical and demographic covariates were explored.

#### Analytical procedures

Normality of the data: Assessed using the Shapiro-Wilk test^25^, with a significance level of p≤0.05.

Homogeneity of variances: Assessed using Levene’s test^26^, prior to parametric intergroup comparisons. Within-group comparisons (pre-*vs*. post-CT): The Wilcoxon signed-rank test^27^ was used to compare pre- and post-CT scores within each group. This nonparametric test was chosen because it does not assume normality and is appropriate for paired samples.

Between-group comparisons (Aβ+, Aβ-, HC):

- One-way ANOVA^28^, followed by Tukey’s post hoc test^29^, for variables with normal distribution and homogeneous variances.
- Kruskal-Wallis test^30^, followed by Dunn-Bonferroni post hoc test^31^, for nonparametric comparisons when assumptions were not met.

#### Dependent variables

Statistical analysis focused on the following cognitive outcome measures:

GTs (GT I, GT II):

Immediate and delayed recall of journalistic reports (total score: 72 points).

Verbal episodic memory:

- (LM I) – immediate recall of episodic memory.
- (LM II) – delayed recall of episodic memory.

RAVLT A1–A5: verbal learning and immediate recall across five acquisition trials.

RAVLT A7: delayed recall after interference.

#### Significance level

Two-tailed alpha level of p≤0.05.

Bonferroni corrections applied when appropriate to control for Type I error in multiple comparisons.

## RESULTS

### Baseline characteristics

Baseline demographic and clinical characteristics of the sample are presented in Table 1. All participants were right-handed. No significant differences were observed between groups regarding age, gender distribution, years of education, estimated IQ, or mood symptoms (Hospital Anxiety and Depression Scale – HADS scores), indicating adequate comparability across groups at baseline.

**Table 1:** Baseline demographic and clinical characteristics.

Variable	HC (n=16)	Aβ-MCI (n=20)	Aβ+MCI (n=19)	Total sample (n=55)
Age in years (mean, SD)	74.2±(8.33)	73.3±(5.89)	73.4±(7.18)	73.6±(6.99)
Female gender (n, %)	14 (87.5%)	14 (70.0%)	15 (78.9%)	43 (78.2%)
Right-handed (n, %)	16 (100.0%)	20 (100.0%)	19 (100.0%)	55 (100.0%)
Education in years (mean, SD)	11.9±(2.92)	12.0±(4.17)	12.2±(6.36)	12.0±(4.69)
Estimated IQ (mean, SD)	98.7±(8.12)	97.7±(12.4)	95.0±(9.09)	97.7±(10.18)
Mood Scale (HADS) (mean, SD)	9.92±(4.35)	9.68±(5.63)	9.50±(4.16)	9.67±(4.48)

Source: The authors.

Abbreviations: HC, healthy controls; MCI, mild cognitive impairment; SD, standard deviation; IQ, Intelligence Quotient; HADS, Hospital Anxiety and Depression Scale.

### Training effects

Pre- and post-training performance was analyzed across the three groups (Aβ+aMCI, Aβ-aMCI, and HC) for episodic memory measures (LM I and II, RAVLT A1–A5 and A7) and GT I and GT II.

### Logical memory

**Figure 2:**
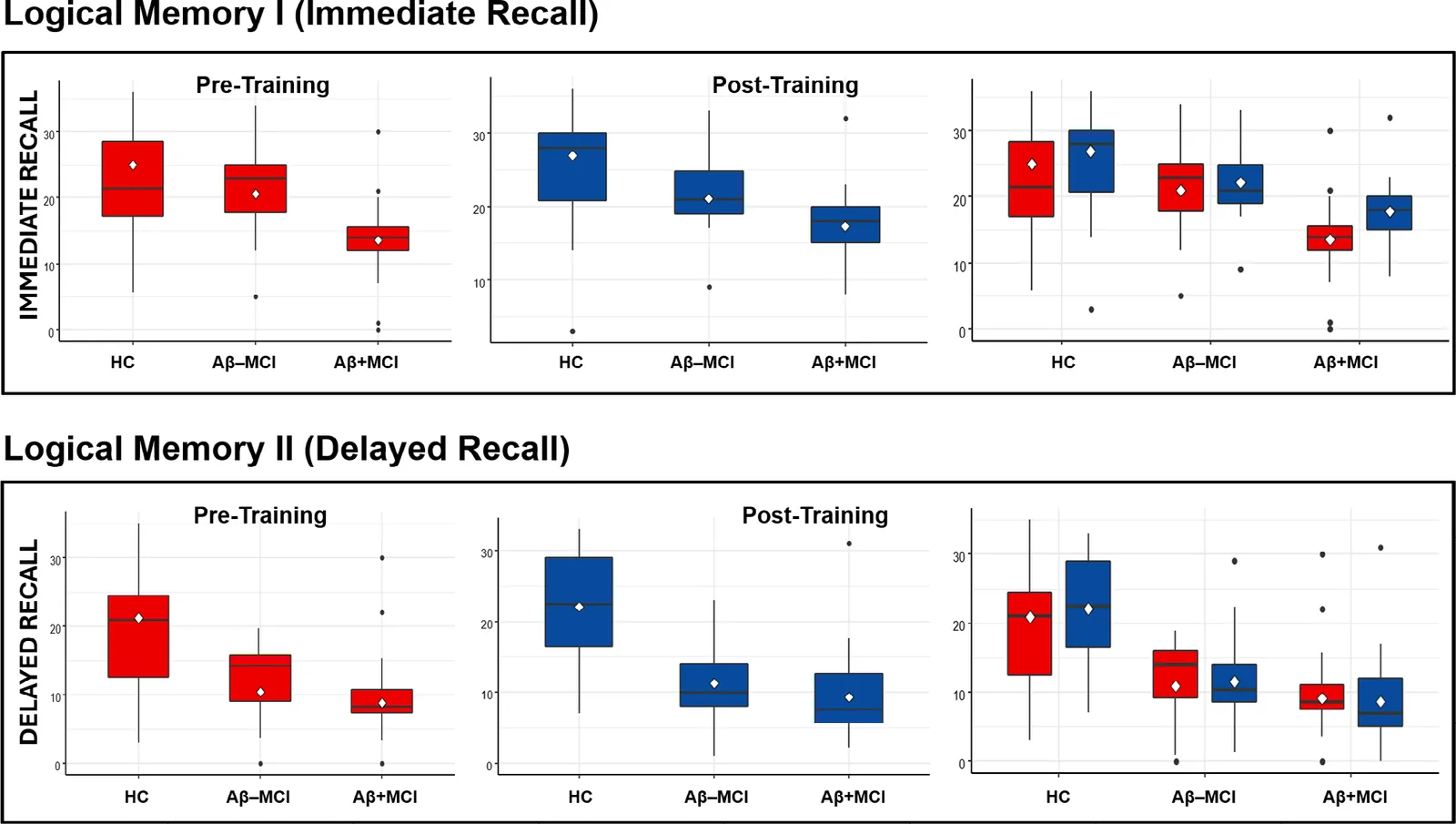
Logical Memory I and II.

Source: The authors.

For LM I, before training, significant group differences were observed (ANOVA-F – F=16.24; p<0.01), with HC showing the highest performance (mean – M=25.25), followed by Aβ-aMCI (M=20.22) and Aβ+aMCI (M=13.37). HC and Aβ-aMCI, while no significant difference was observed between HC and Aβ-aMCI (Tables 2 and 3; Figure 2). Post-training, all groups showed increased scores, and the group effect remained significant (F=9.101; p<0.01). Post hoc comparisons indicated significant differences between HC and both Aβ+aMCI and Aβ-aMCI, whereas no significant difference was observed between Aβ+aMCI and Aβ-aMCI (Tables 2 and 3; Figure 2).

**Table 2:** Results of the Groups - Pre-training and Post-training.

Comparation in same Group between Pre- and post-training (∆ of mean in same group)				Comparation between Groups (∆ of mean between different Groups; p=value)		
Variable	HC	Aβ-aMCI	Aβ+aMCI	HC vs.	HC vs.	Aβ-aMCI vs.
	(n=16)	(n=20)	(n=19)	Aβ+aMCI	Aβ-aMCI	Aβ+aMCI
Generalization Task (GT I e GT II) – mean; (SD; median) – 95%CI						
Pre-training	48.88 (12.20–53)	44.53 (9.01–45)	34.73 (14.37–37)	14.15 p=0.0043	4.35 p=0.4740	9.8 p=0.0043
Post-training	54.40 (9.79–54)	47.71 (11.31–52)	38.08 (14.98–39)	16.32 p=0.0027	6.69 p=0.3715	9.63 p=0.1067
∆ Pre x Post	+5.52	+3.18	+3.35	Outcome: HC>Aβ+aMCI>Aβ-aMCI		
Logical I – Immediate Memory - mean; (SD; median) – 95%CI						
Pre-training	25.25 (7.35–25)	20.22 (4.19–22)	13.37 (6.73–14.5)	11.88 p<0.01	5.03 p=0.057	6.85 p<0.01
Post-training	27.07 (6.56–28)	21.00 (6.10–21)	17.50 (5.9–18)	9.57 p<0.01	6.07 p=0.027	3.5 p=0.255(NS)
∆ Pre x Post	+1.82	+0.78	+4.13	Outcome: Aβ+aMCI>HC>Aβ-aMCI		
Logical Memory II – Delayed Recall – mean; (SD; median) – 95%CI						
Pre-training	21.31 (8.18–21)	10.78 (2.65–16)	8.74 (4.24–13)	12.57 p<0.01	10.53 p<0.01	2.04 p=0.361
Post-training	22.14 (7.40–21)	11.50 (3.58–16)	9.50 (4.44–11)	12.64 p<0.01	10.64 p=0.01	2.00 p=0.387(NS)
∆ Pre x Post	+0.83	+0.72	+0.76	Outcome: HC>Aβ+aMCI>Aβ-aMCI		
RAVLT A1–A5 – Immediate Verbal Recall - mean; (SD; median) – 95%CI						
Pre-training	44.31 (7.34–44)	38.22 (9.15–39)	36.74 (9.61–37)	7.77 p=0.038	6.09 p=0.121(NS)	1.48 p=0.866(NS)
Post-training	51.36 (6.11–51)	40.69 (8.46–41.5)	40.25 (8.52–38)	11.11 p<0.01	10.67 p<0.01	0.44 p=0.986(NS)
∆ Pre x Post	+7.05	+2.47	+3.51	Outcome: HC>Aβ+aMCI>Aβ-aMCI		
RAVLT A7 – Delayed Verbal Recall – mean; (SD; median) – 95%CI						
Pre-training	11.44 (3.08–10)	7.05 (2.55–7)	5.89 (2.18–7)	5.55 p<0.01	4.39 p<0.01	1.16 p=0.363(NS)
Post-training	11.43 (2.21–11)	7.88 (2.16–7.5)	6.69 (3.42–8)	4.74 p<0.01	3.55 p<0.01	1.19 p=0.429(NS)
∆ Pre x Post	**-0.01 (stable)**	**+0.83**	**+0.80**	Outcome: Aβ-aMCI>Aβ+aMCI>HC		

Source: The authors.

Abbreviations: HC, healthy controls; aMCI, amnestic mild cognitive impairment; GT, generalization tasks; SD, standard deviation; CI, confidence interval; NS, not significant.

**Table 3:** Logical Memory I (Immediate Recall) and Logical Memory II (Delayed Recall) between-group comparison.

Logical Memory I (Immediate Recall)	
Pre-training^*^	Post-training^*^
F=1.291, p=0.284 → homogeneity confirmed	F=0.248, p=0.78 → homogeneity confirmed
^ †^F(2, N)=16.24, p<0.01	^ †^F(2, N)=9.101, p<0.01
• HC *vs*. Aβ+aMCI: p<0.01	• HC *vs*. Aβ+aMCI: p<0.01
• HC *vs*. Aβ-aMCI: p=0.057	• HC *vs*. Aβ-aMCI: p=0.027
• Aβ-aMCI *vs.* Aβ+aMCI: p<0.01	•Aβ -aMCI *vs.* Aβ+aMCI: p=0.255
**Logical Memory II (Delayed Recall)**	
Pre-training^*^	Post-training^*^
F=5.294, p<0.01 → heteroscedasticity present	F=5.296, p<0.01 → heteroscedasticity present
^ ‡^H(2)=22.814, p<0.01	^ †^F(2, df)=22.122, p<0.01
• HC *vs*. Aβ+aMCI: p<0.01	• HC *vs*. Aβ-aMCI: p<0.01
• HC *vs.* Aβ+aMCI: p<0.01	• HC *vs.* Aβ-aMCI: p=0.01
• Aβ-aMCI *vs*. Aβ+aMCI: p=0.361	• Aβ-aMCI *vs.* Aβ+aMCI: p=0.387

Notes: ^*^Post hoc (Kruskal-Wallis);

^†^One-way ANOVA;

^‡^Post hoc (Tukey).

Abbreviations: HC, healthy controls; aMCI, amnestic mild cognitive impairment. Source: The authors.

For LM II, before training, significant group differences were observed (H=22.814; p<0.01), with HC showing higher performance (M=21.31) compared to both Aβ-aMCI (M=10.78) and Aβ+aMCI (M=8.74). Post hoc comparisons indicated significant differences between HC and both Aβ-aMCI and Aβ+aMCI, while no significant difference was observed between Aβ-aMCI and Aβ+aMCI (Tables 2 and 3; Figure 2). Post-training, all groups showed slight increases, and the group effect remained significant (F=22.122; p<0.01), with HC differing from both Aβ-aMCI and Aβ+aMCI, while no significant difference was observed between Aβ-aMCI and Aβ+aMCI (Tables 2 and 3; Figure 2).

### RAVLT

**Figure 3:**
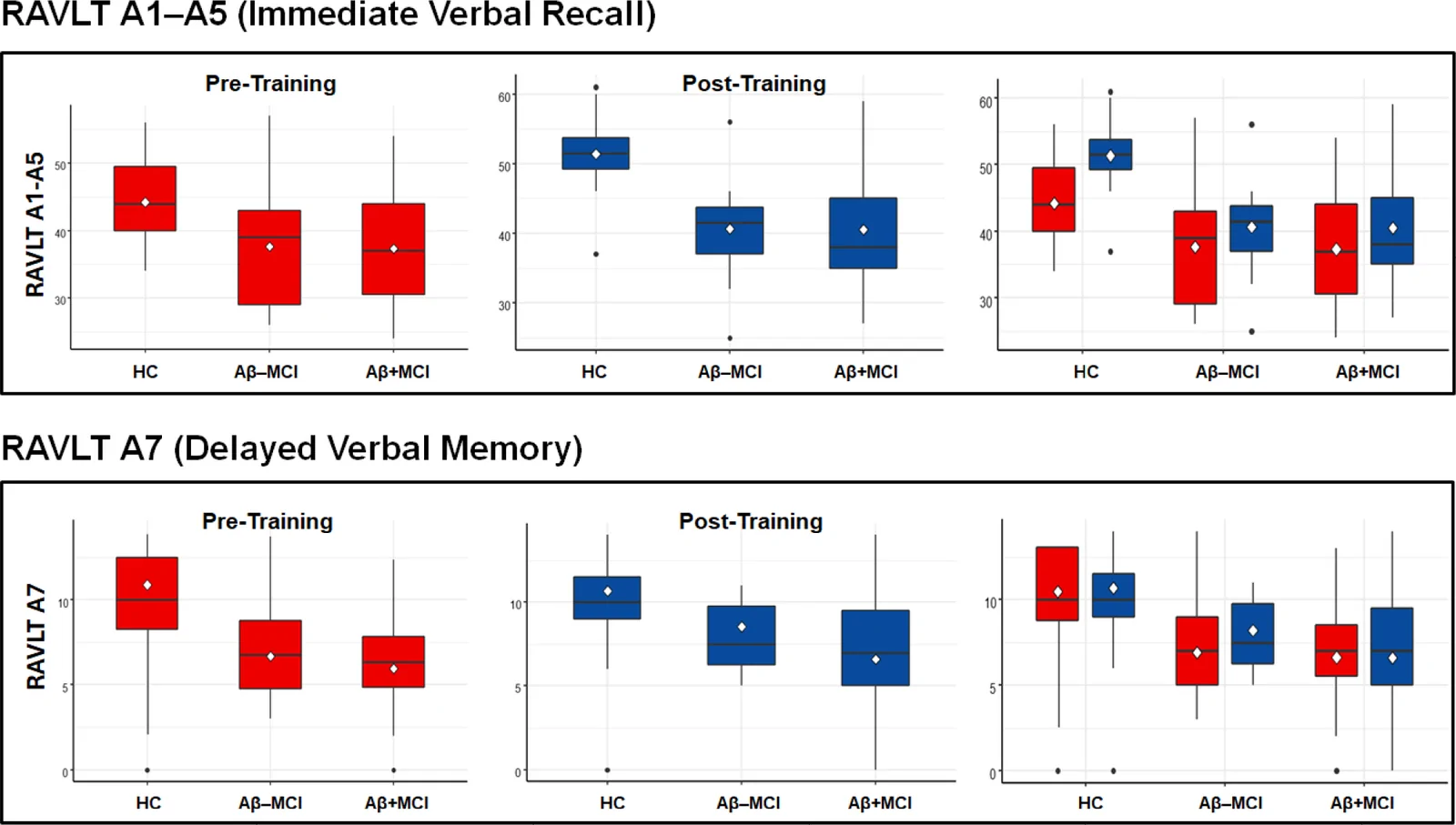
RAVLT A1–A5 and RAVLT A7.

Source: The authors.

For RAVLT immediate recall (A1-A5), before training, significant group differences were observed (F=3.50; p=0.038), with HC showing higher performance (M=44.31) compared to Aβ+aMCI. Post hoc comparisons indicated a significant difference between HC and Aβ+aMCI, while no significant differences were observed between HC and Aβ-aMCI, nor between Aβ-aM-CI and Aβ+aMCI (Tables 2 and 4; Figure 3). Post-training, all groups showed improvement (HC: +7.05; Aβ-aMCI: +2.47; Aβ+aMCI: +3.51), with a persistent group effect (F=9.38; p<0.01). Post hoc comparisons indicated significant differences between HC and both Aβ-aMCI and Aβ+aMCI, while no significant difference was observed between Aβ-aMCI and Aβ+aMCI (Tables 2 and 4; Figure 3).

**Table 4:** Rey Auditory Verbal Learning Test A1–A5 (Immediate Verbal Recall) and A7 (Delayed Verbal Memory) between-group comparison.

RAVLT A1–A5 (Immediate Verbal Recall)	
Pre-training^*^	Post-training^*^
F=0.628, p=0.538 → variances homogeneous	F=1.067, p=0.353 → variances homogeneous
^†^F(2, N)=3.50, p=0.038 → significant difference	^†^F(2, N)=9.38, p<0.01 → significant difference
•HC *vs*. Aβ+aMCI: p=0.038	•HC *vs.* Aβ-aMCI: p=0.121 (NS)
•HC *vs.* Aβ-aMCI: p<0.01	•HC *vs.* Aβ+aMCI: p<0.01
•Aβ-aMCI *vs*. Aβ+aMCI: p=0.866	•Aβ-aMCI *vs.* Aβ+aMCI: p=0.986
**RAVLT A7 (Delayed Verbal Memory)**	
Pre-training^*^	Post-training^*^
F=1.787, p=0.178 → variances are homogeneous	F=2.545, p=0.090
^ †^F(2, df)=21.470, p<0.01 → significant difference	^ †^F(2, df)=12.46, p<0.01 → significant difference
•HC *vs*. Aβ+aMCI: p<0.01	•HC *vs.* Aβ-aMCI: p<0.01
•HC *vs.* Aβ-aMCI: p<0.01	•HC *vs.* Aβ+aMCI: p<0.01
•Aβ-aMCI *vs*. Aβ+aMCI: p=0.363	•Aβ-aMCI *vs.* Aβ+aMCI: p=0.429

Notes: ^*^Post hoc (Tukey);

^†^One-way ANOVA.

Abbreviations: HC, healthy controls; aMCI, amnestic mild cognitive impairment.

Source: The authors.

For RAVLT delayed recall (A7), before training, significant group differences were observed (F=21.470; p<0.01), with HC showing higher performance (M=11.44) compared to both Aβ-aMCI (M=7.05) and Aβ+aMCI (M=5.89). Post hoc comparisons indicated significant differences between HC and both Aβ-aMCI and Aβ+aMCI, while no significant difference was observed between Aβ-aMCI and Aβ+aMCI (Tables 2 and 4; Figure 3). Post-training, modest increases were observed in Aβ-aMCI and Aβ+aMCI, while HC remained stable. The group effect remained significant (F=12.46; p<0.01), with post hoc comparisons indicating significant differences between HC and both Aβ-aMCI and Aβ+aMCI, and no significant difference between Aβ-aMCI and Aβ+aMCI (Tables 2 and 4; Figure 3).

### Generalization tasks

**Figure 4:**
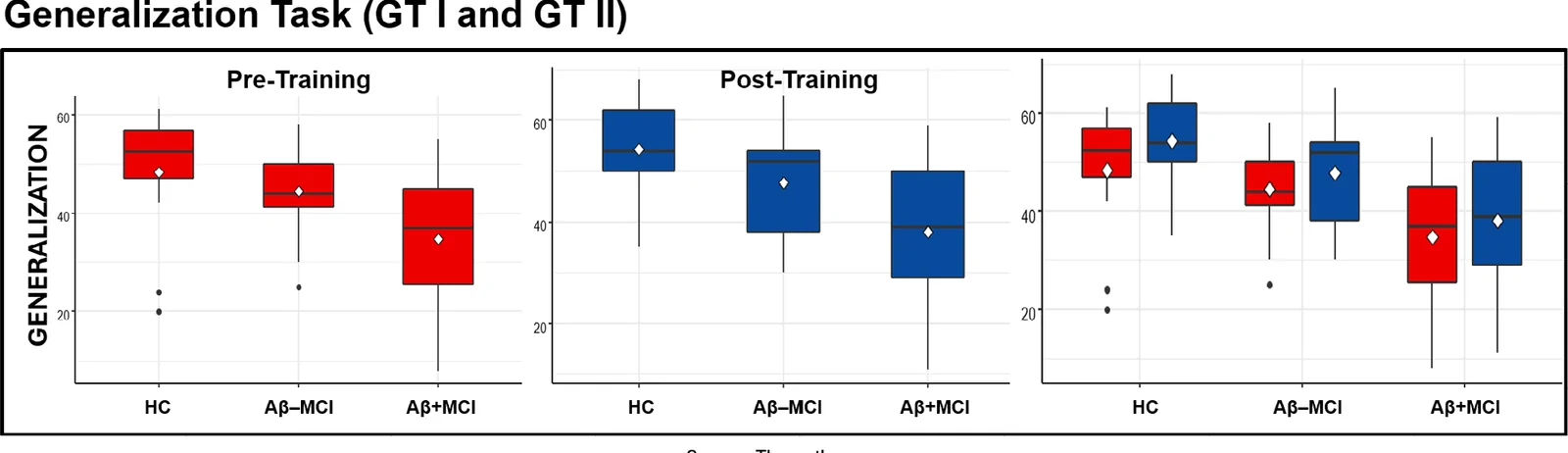
Generalization tasks.

Source: The authors.

Before training, significant group differences were observed (H=10.176; p=0.0061), with lower performance shown in the Aβ+aMCI group (M=34.73, SD=14.37) compared to HC (M=48.88, SD=12.20). Significant differences were observed between Aβ+aMCI and both HC and Aβ-aMCI, while no significant difference was observed between HC and Aβ-aMCI (Tables 2 and 5; Figure 4). Post-training, all groups showed increased scores (HC=54.40, SD=9.79; Aβ-aMCI=47.71, SD=11.31; Aβ+aMCI=38.08, SD=14.98). A significant group effect remained (H=6.435; p=0.0036), with differences observed between HC and Aβ+aMCI, while no significant differences were found between Aβ-aMCI and the other groups (Tables 2 and 5; Figure 4).

**Table 5:** Generalization Task between-group comparison.

Generalization Task (GT I and GT II)	
GT I – Pre-training^*^	GT II – Post-training^*^
F=1.5838, p=0.2164	F=1.1523, p=0.3257
• Statistical Test Value: 10.176, df: 2, p=0.0061	• Statistical Test Value: 6.435, df: 2, p=0.0036
•HC *vs.* Aβ+aMCI: p=0.0043 •HC *vs.* Aβ-aMCI: p=0.4740 •Aβ-aMCI *vs.* Aβ+aMCI: p=0.0043	•HC *vs.* Aβ+aMCI: p=0.0027 •HC *vs.* Aβ-aMCI: p=0.3715 •Aβ-aMCI *vs.* Aβ+aMCI: p=0.1067

Note: ^*^Post hoc (Kruskal-Wallis).

Abbreviations: GT, generalization tasks; HC, healthy controls; aMCI, amnestic mild cognitive impairment; df, degress of freedom.

Source: The authors.

## DISCUSSION

The present study investigated the effects of a cognitive training protocol based on mental imagery strategies in individuals with aMCI, stratified according to amyloid status (Aβ+ and Aβ-), as well as in HC. The results demonstrated significant improvements in immediate episodic recall and performance on generalization tasks across all groups, whereas gains in delayed recall were more modest.

Measures of immediate recall memory (RAVLT A1– A5 and LM I) assess encoding efficiency and learning processes, whereas measures of delayed recall memory (RAVLT A7 and LM II) specifically assess consolidation-dependent processes. The differential pattern of improvement observed, characterized by greater gains in immediate recall compared with delayed recall, indicates that the intervention primarily acted on encoding-related processes, with a more limited effect on consolidation mechanisms. This interpretation is consistent with evidence demonstrating that explicit mnemonic strategy training can selectively enhance encoding efficiency in individuals with aMCI. Hampstead et al.^32^ demonstrated that structured training based on visual imagery and semantic elaboration significantly improves associative memory performance (face–name pairs), supporting the notion that strategic encoding processes remain amenable to modification despite the underlying cognitive impairment.

From a neurocognitive perspective, this dissociation between immediate and delayed recall may be interpreted in light of the neural systems underlying different stages of memory processing. Measures of immediate recall, such as RAVLT A1–A5 and LM I, primarily depend on encoding and initial learning processes and involve distributed cortical networks, including prefrontal and temporoparietal regions associated with organization, semantic processing, and verbal information integration^33,34^. In contrast, measures of delayed recall, such as RAVLT A7 and LM II, are more directly related to consolidation and episodic retrieval processes, which depend on the integrity of the hippocampal system and medial temporal lobe structures. Evidence indicates that structural and functional alterations in these regions are directly associated with episodic memory performance in individuals with MCI^35,36^. Accordingly, the pattern observed in the present study is consistent with the hypothesis of relative preservation of neocortical networks involved in encoding, in contrast to the greater vulnerability of medial temporal lobe structures throughout the course of neurodegenerative disease.

This interpretation is further supported by the findings related to amyloid status. Despite the lower baseline performance observed in the Aβ+ group, responsiveness to cognitive training was comparable to that observed in the other groups, particularly in measures of immediate recall, suggesting that even in the presence of amyloid pathology, neocortical networks involved in information encoding and organization remain functionally recruitable and amenable to modulation through intervention. From the perspective of cognitive reserve models, these findings may reflect the persistence of compensatory mechanisms capable of supporting learning and strategic memory processes despite the underlying neuropathological burden. Cognitive reserve is conceptualized as the brain’s ability to actively recruit alternative neural networks or employ more efficient processing strategies to compensate for structural damage, thereby delaying the clinical expression of cognitive impairment. In this context, the preserved responsiveness to training observed in the Aβ+ group is consistent with the notion that alternative neural resources may remain functionally available throughout the course of neurodegenerative disease, allowing engagement in strategic learning processes despite established amyloid pathology. Although cognitive reserve was not directly assessed, the mean educational attainment of approximately twelve years observed in the sample is frequently considered one of its principal indirect indicators. Thus, reserve-related mechanisms, such as greater efficiency in recruiting neocortical networks involved in encoding, may have contributed to the preservation of responsiveness to strategy-based interventions even in the presence of underlying amyloid pathology. In contrast, the smaller magnitude of gains observed in delayed recall measures among Aβ+ participants is consistent with evidence indicating that β-amyloid accumulation is associated with early alterations in the medial temporal lobe and hippocampus, disproportionately affecting consolidation-dependent processes^2,4,14,35,36^. Taken together, these findings suggest that amyloid status may differentially influence responsiveness to cognitive training, exerting a greater impact on consolidation-dependent processes than on those related to encoding.

The results indicate not only absolute improvements in performance but also a reduction in between-group differences following the intervention in specific measures, particularly immediate episodic recall, suggesting that cognitive training may contribute to bringing the performance of individuals with MCI closer to that observed in healthy controls, at least with respect to encoding-related processes. Although improvements were observed across all groups, the group effect remained significant for the GTs, LM I and II, and the RAVLT measures (A1–A5 and A7), indicating that between-group differences persisted after the intervention. Nevertheless, the attenuation of these differences in specific measures, particularly LM I, suggests a pattern of partial convergence in performance restricted to encoding-related processes. The greater gains observed in tasks involving structured narrative material, such as LM, compared with word-list learning tasks such as the RAVLT, suggest that the effectiveness of the intervention may depend on the organization of the material being learned, with more pronounced effects when the content facilitates the construction of integrated episodic representations.

The gains observed in the generalization tasks, even in the absence of explicit instructions to apply the learned strategy, indicate spontaneous transfer of learning, a central aspect of the ecological validity of cognitive interventions. Because these tasks required participants to encode and recall novel newspaper articles addressing everyday topics, they constitute a measure of strategy application with greater ecological relevance than traditional neuropsychological memory tests. Although the present study was designed to assess cognitive rather than functional outcomes, the findings suggest that participants were able to apply the trained strategy beyond the specific training context. However, because functional outcomes, subjective memory complaints, quality of life, and disease progression were not directly assessed, the functional relevance of this transfer remains an important question for future research.

In contrast to the findings observed in the generalization tasks and measures of immediate recall, gains in delayed recall were more limited, reinforcing the notion that consolidation-dependent processes may be less responsive to short-term cognitive interventions, particularly in populations with impairment of the neural systems involved in episodic memory consolidation. These findings are consistent with evidence from meta-analyses demonstrating that cognitive training is associated with small to moderate improvements in memory-related outcomes in individuals with MCI, although substantial methodological heterogeneity exists across studies^37,38^. More specifically, this body of evidence suggests that interventions incorporating explicit mnemonic strategies tend to produce more consistent effects on learning and episodic encoding measures, whereas effects on delayed recall, which is more dependent on consolidation processes, are generally more modest and heterogeneous across studies^37,38,39^. This pattern is compatible with the hypothesis that consolidation-dependent processes, because they require greater integrity of the neural systems involved in the formation and stabilization of episodic memories, may exhibit reduced responsiveness to short-duration cognitive interventions.

With regard to empirical studies, the findings of the present investigation partially converge with those reported by Belleville et al.^9^, who observed improvements in delayed recall and associative memory following a multimodal cognitive training program. However, the results reported here differ with respect to the magnitude of effects on delayed memory, which were more limited. This discrepancy may be related to differences in intervention protocols, the nature of the materials employed, and the cognitive processes predominantly engaged by the training. Hampstead et al.^10,11^ demonstrated improvements in memory performance accompanied by functional brain changes following explicit mnemonic strategy training, supporting the notion that strategy-based interventions can modulate neural networks associated with memory. Convergent findings have also been reported by Rosen et al.^33^, Balardin et al.^34^, and Carlson et al.^40^, who observed improvements in memory performance associated with modulation of frontoparietal and associative networks. Consistent with these findings, Hwang et al.^41^ reported cognitive gains in both individuals with aMCI and patients with early-stage AD, indicating responsiveness to cognitive training across different clinical stages. Nevertheless, the smaller magnitude of effects observed in more advanced stages, as described by these authors, is consistent with the limited impact on consolidation-dependent processes observed in the present study.

The absence of a non-intervention control group represents an important limitation and prevents definitive attribution of the observed improvements exclusively to the cognitive training protocol, as repeated exposure to neuropsychological measures may have contributed, at least in part, to the gains observed. Nevertheless, the pattern of results does not appear to be fully compatible with a generalized practice effect. If the improvements had been predominantly driven by familiarity with the assessment instruments, a more homogeneous pattern of gains would be expected across cognitive measures and participant groups. Instead, improvements were selective and occurred predominantly in measures related to the cognitive processes specifically targeted by the intervention, whereas other measures showed more modest or nonsignificant changes. Furthermore, the improvements observed in the generalization tasks, which employed novel materials not previously presented during either training or assessment, provide preliminary evidence of learning transfer beyond simple test familiarity. Even so, future studies incorporating non-intervention control groups will be important to more precisely distinguish the specific effects of training from those potentially related to repeated assessment.

Several considerations should be taken into account when interpreting the present findings. The sample was composed of participants with a mean educational level of approximately twelve years, a characteristic that may be relevant for contextualizing the responsiveness observed following cognitive training, particularly among individuals with amyloid pathology. Although cognitive reserve was not directly assessed, educational attainment is frequently considered one of its principal indirect indicators. Accordingly, reserve-related mechanisms may have contributed to the preservation of learning capacity and responsiveness to strategy-based interventions despite the presence of underlying neuropathological burden.

Another consideration concerns the relatively modest sample size, which reflects both the complexity of the study protocol and the characteristics of the population under investigation. Details regarding blinding procedures, group allocation, and sample constitution are provided in the Methods section. In addition, the intervention was specifically designed to examine the effects of a mental imagery strategy on episodic memory processes. For this reason, the training protocol was intentionally limited to six sessions, allowing a more focused evaluation of the strategy under investigation, although a greater number of sessions might produce additional benefits.

Finally, the study was not designed to directly evaluate functional outcomes, quality of life, autonomy in activities of daily living, or disease progression. The inclusion of generalization tasks based on the encoding and recall of novel newspaper articles resembling everyday informational demands provides preliminary evidence that the benefits of training may extend beyond the specific context in which the strategy was taught. Future studies incorporating longer intervention periods, longitudinal follow-up, and direct functional outcome measures will be important to more comprehensively clarify the clinical applicability and long-term impact of these cognitive gains.

In conclusion, the present study demonstrated that a cognitive training protocol based on mental imagery strategies is associated with improved episodic memory performance in individuals with aMCI, regardless of amyloid status, as well as in cognitively healthy older adults. The pattern of results observed indicates that the intervention acted predominantly on encoding and learning processes, producing more limited effects on delayed recall.

Although between-group differences remained significant across multiple measures, the attenuation of these differences in specific tasks, particularly those involving structured material, suggests that cognitive training may contribute to partially reducing performance disparities associated with both clinical diagnosis and biomarker status, especially at the level of encoding-related processes. The responsiveness to training observed in the Aβ+ group, despite lower baseline performance, indicates preservation of the functional capacity to engage in learning processes even in the presence of underlying neuropathology. These findings suggest that compensatory mechanisms may support responsiveness to strategy-based interventions throughout the course of neurodegenerative disease and support the potential applicability of such interventions during these clinical stages. The limited effects observed in delayed recall, however, highlight constraints related to consolidation-dependent processes, suggesting that such mechanisms may be less responsive to short-duration cognitive interventions.

Taken together, these findings contribute to understanding both the potential and the limitations of cognitive training in MCI, suggesting that its effects are more pronounced for encoding processes than for consolidation mechanisms and may vary according to task characteristics and the integrity of the underlying neural systems.

## Data Availability

The datasets generated and/or analyzed during the current study are not publicly available due to ethical/legal/privacy restrictions but are available from the corresponding author upon reasonable request.
